# Is microwave ablation therapy as effective as colorectal liver metastases in noncolorectal liver metastases?

**DOI:** 10.55730/1300-0144.5440

**Published:** 2022-06-04

**Authors:** Gürkan DANIŞAN, Ali KÜPELİ, Ogün TAYDAŞ

**Affiliations:** 1 Department of Radiology, Faculty of Medicine, Sakarya University, Sakarya, Turkey; 2 Department of Radiology, Faculty of Medicine, Trabzon Kanuni Training and Research Hospital, Trabzon, Turkey; 3 Department of Family Medicine, Haydarpasa Numune Training and Research Hospital, İstanbul, Turkey

**Keywords:** Microwave ablation, metastasis, liver, ultrasound, thermal ablation

## Abstract

**Background/aim:**

The aim of the study was to evaluate the relationship between primary tumor type and the effectiveness of microwave ablation (MWA) therapy by comparing the technical and clinical outcomes of MWA in the treatment of colorectal liver metastases (CLM) and noncolorectal liver metastases (NCLM).

**Materials and methods:**

Between January 2019 and March 2021, 47 consecutive patients (25 male, 22 female) with a total of 63 unresectable hepatic metastases underwent MWA under ultrasound guidance. The patients were divided into CLM (n = 29) and NCLM (n = 18) groups. Patient demographics, procedural details, and complications were noted. The overall survival (OS) and disease-free survival (DFS) rates were also analyzed.

**Results:**

Technical success was 100% in both groups. No major complication was observed. Three minor complications [fatigue (n = 2) and subcutaneous hematoma (n = 1)] were encountered. DFS rates were 88.9%, 71.9%, 64.9%, and 44.0% at 3, 6, 12, and 24-months, respectively, with a mean DFS of 17.4 months (95% CI: 15.1, 19.7). Also, OS rates were 93.7%, 90.0%, 76.8%, and 64.3%, at 3, 6, 12, and 24-months, respectively, with a mean OS of 18.5 months (95% CI: 16.2, 20.7). There was no significant difference in recurrence between the CLM and NCLM groups (p = 0.452). The recurrence rate in liver metastases > 3 cm in size was significantly higher than in metastases ≤ 3 cm in size (p < 0.001).

**Conclusions:**

MWA therapy is as effective in the NCLM group as in the CLM group, regardless of histologic type. Metastasis size (>3 cm) was correlated with the recurrence rate in the CLM and NCLM groups.

## 1. Introduction

Though metastatic liver tumors are associated with increased mortality and morbidity, clinical outcomes of patients with liver metastases improved greatly in recent years [1]. After lymph nodes, the liver is the second most common organ with metastases [2]. The most common metastases to the liver are caused by colorectal cancer (59%), with more than one segment being affected in 80% of cases [3]. Surgery is the gold standard treatment method in liver metastasis; however, it can only be performed in 20% of metastatic liver lesions [4,5]. The majority of liver malignancies cannot be surgically treated due to the presence of comorbid diseases, multiple metastases, anatomic localization precluding resection, insufficient functional liver capacity, and tumor recurrence [6,7].

Many physicians and patients also prefer interventional therapies due to the lower mortality and morbidity risks compared to surgery [8]. The combination of thermal ablation therapies with surgical resection offers a high cure probability in eligible patients [9]. Various ablation methods have been developed, including radiofrequency ablation (RFA) and microwave ablation (MWA), and both can be done percutaneously with imaging guidance [10]. Compared to RFA, MWA has several advantages such as shorter ablation times, larger ablation areas, the lower heat sink effect, and no need for grounding pads [11].

Percutaneous MWA is a safe and effective method in the treatment of both primary and metastatic hepatic malignancies that are not suitable for surgery [12]. Also, high success rates have been reported in the treatment of MWA up to 5.5 cm in colorectal tumor metastases [13]. However, there are few studies on the efficacy of MWA therapy in noncolorectal liver metastases [14]. This study aims to evaluate the relationship between primary tumor type and the effectiveness of microwave ablation (MWA) therapy by comparing the technical and clinical success of MWA therapy for colorectal liver metastases (CLM) and noncolorectal liver metastases (NCLM).

## 2. Materials and methods

### 2.1. Participants

This retrospective study was conducted in compliance with the principles of the Helsinki declaration, and informed consent was obtained from each patient. Ethical approval for this retrospective study was obtained from the Institutional Review Board (2021-21460-217). Patients with metastatic liver disease who were referred to our clinic for thermal ablation between January 2019 and March 2021 were included in the study after obtaining their informed consent.

An oncology committee consisting of radiologists, oncological surgeons, gastroenterologists, medical oncologists, radiation oncologists, and nuclear medicine specialists made all decisions about MWA therapy on a multidisciplinary. Inclusion criteria were as followed; (i) ineligible for surgery due to comorbidities, (ii) anatomical localization where surgical treatment is not feasible or safe, (iii) insufficient functional liver capacity after surgery (especially in recurrent surgical procedures), (iv) the patient’s refusal to undergo surgery. Exclusion criteria were; (i) presence of extrahepatic metastases, (ii) pregnancy, (iii) uncorrectable coagulopathy.

A total of 47 patients with 63 lesions met the above criteria and were enrolled in this study. The patients were divided into two groups as CLM and NCLM. Therewithal, to determine how tumor size affected the recurrence rates, the lesions were classified into two groups: ≤3 cm lesions and >3 cm lesions. Also, the location of liver metastases was noted.

### 2.2. Ablation procedures

**Figure 1:**
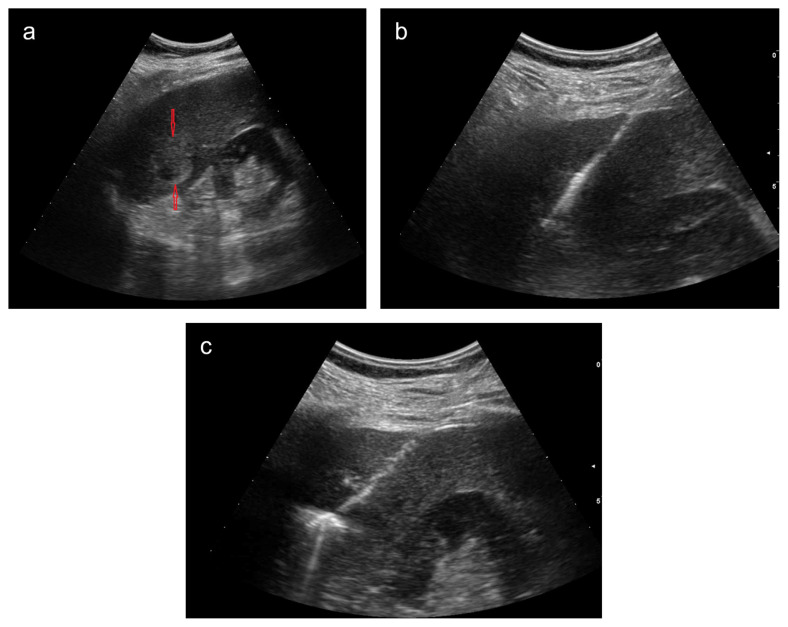
Ultrasound images of a 61-year-old male patient show a 35 mm hyperechoic colon cancer metastasis in liver segment 6 (red arrows) (a), placement of the microwave ablation probe in the metastasis (b), and ablation process (c).

One interventional radiologist with 7-years’ experience on interventional oncology performed all procedures. MWA was performed in all patients under deep sedoanalgesia under the supervision of an anesthesiologist. Under sterile conditions, local anesthesia was applied to the subcutaneous area and the liver capsule with 10 cc of prilocaine (Priloc 2%, Vem Pharmaceuticals, Turkey). Lesions were accessed under ultrasound guidance (Esaote MyLab Seven, China) with a convex probe. The Eco (Nanjing Eco System, China) device, which works with a frequency of 2.45 GHz, is shaft-cooled and continuous energy transferring, was used for MWA. Considering the location and size of the lesion, 14-16-17-gauge MWA antennas were used. Similarly, the ablation procedure was performed at varying power and duration depending on the size of the lesion. Successful treatment was defined as ablation of the tumor with a 10 mm margin, preserving intact parenchyma and nontarget tissues [15] (Figure 1). In 21 patients, additional hydrodissection with 0.9% saline solution was required for the lesions that were close to the bowel loops, diaphragm, and large vessels (if closer than 5 mm) [16]. The technical success of MWA was defined as the depiction of complete ablation at the multiphasic CT performed one day after the procedure.

### 2.3. Evaluation of tumor response

The modified Response Evaluation Criteria in Solid Tumors (mRECIST) were used to evaluate the ablation procedure. Adverse events and complications after treatment are classified according to the SIR (Society of Interventional Radiology) adverse event classification [17]. The local tumor progression (LTP) was described as the detection of nodular enhancement in the adjacent ablation zone during follow-up.

### 2.4. Follow-up of patients

**Figure 2:**
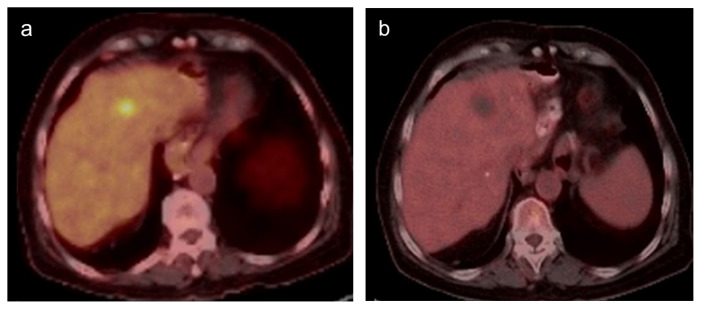
PET CT images of a 61-year-old female patient show gastric cancer metastasis in liver segment 4A (a) and no metabolic activity in ablation zone consistent with complete response after microwave ablation therapy (b).

**Figure 3:**
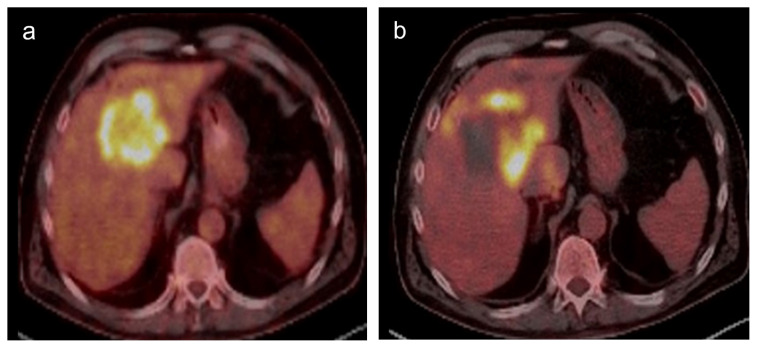
PET CT images of a 60-year-old male patient show colon cancer metastasis in liver segment 4A-8 junction level (a) and metabolic activity after microwave ablation therapy in the edge of ablation zone consistent with recurrence (b).

The multiphasic computed tomography (CT) (64-row multidetector CT, Aquilion 64; Toshiba Medical Systems, USA) examination was performed every three months for the first year and then every six months thereafter. The postprocedural ablation zone diameter ratio to preprocedural tumor diameter was calculated on CT images one day after the procedure. Follow-up imaging was performed with multiphasic CT or PET CT (Figures 2–3). If the CT findings were equivocal, magnetic resonance imaging and/or PET-CT were performed for confirmation.

### 2.5. Statistical analysis

Statistical analyzes were performed with the SPSS 13.0 Statistical Software (SPSS Inc., Chicago, IL, USA). As statistical analysis, for descriptive statistics, categorical variables were presented with the number, percent, and continuous variables with mean ± standard deviation and median (minimum and maximum) values. Pearson’s chi-square test was used for the comparison of categorical variables. Continuous variables were compared with nonparametric (Mann-Whitney U test) and parametric (Student’s t-test) methods according to their conformity to normal distribution evaluated using the Kolmogorov-Smirnov tests. The Kaplan–Meier survival curves were used for survival analysis. The statistical significance level was accepted as p < 0.05.

## 3. Results

The data of 39 lesions in 29 patients (22 males, 7 females) with CLM and 24 lesions in 18 patients (3 males, 15 females) with NCLM were analyzed. The characteristics of the patients are shown in Table 1. Complete ablation was observed in all lesions in control CT one day after the ablation procedure. The mean age of the patients with CLM and patients with NCLM were 64.1 ± 8.9 and 50.7 ± 9.2 years, respectively. The mean age was significantly higher in the patients with CLM than in the patients with NCLM (p < 0.001). The primary tumors of the patients are presented in Table 2. Six patients had two metastatic lesions, and five patients had three metastatic lesions. The mean lesion sizes were 27.2 ± 15.8 mm and 23.7 ± 12.3 mm for CLM and NCLM groups, respectively. There was no statistically significant difference between the two groups in terms of lesion sizes (p = 0.163).

There were five (7.9%) lesions located near large vessels, three (4.8%) lesions located close to the gastrointestinal tract, six (9.5%) lesions adjacent to the diaphragm, five (7.9%) lesions located near the liver capsule and two (3.2%) lesions located near the gallbladder. The hydrodissection was performed on 21 lesions.

A 16-G, 14-G, and 17-G antennas were used in 44 (69.8%), 12 (19.0%), and 7 (11.2%) lesions, respectively. The mean applied power was 40 ± 4.4 watts. The mean ablation time was 4 ± 1.6 min. The ratio of the diameter of the ablation zone to the tumor diameter was 1.72 ± 0.56 in lesions with no recurrence and 1.37 ± 0.45 in lesions with recurrence (p = 0.173).

Three patients had grade A complications according to the SIR adverse event classification (6.4%). Two of these patients had fatigue for 2 days after the MWA procedure. A subcutaneous hematoma was encountered at the insertion site of the ablation probe, which did not require transfusion and resolved spontaneously.

The mortality rate was 24.1% (7 of 29 patients) in the CLM group; the causes of death for patients were extrahepatic metastases (n = 4), cardiovascular events (n = 2), and local tumor progression (n = 1). Also, the mortality rate was 22.2% (4 of 18 patients) in the NCLM group; the causes of death for patients were extrahepatic metastases (n = 2), cerebrovascular event (n = 1), and local tumor progression (n = 1).

**Figure 4:**
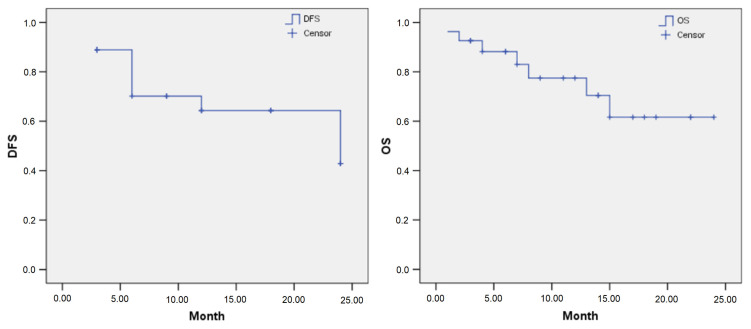
Disease-free survival (DFS) and overall survival (OS) in hepatic metastases.

The median follow-up time was 9 (range 3–24) months. The 3-, 6-, 12-, 24- months disease free survival (DFS) rates were 88.9%, 71.9%, 64.9% and 44.0%, respectively, with a mean DFS of 17.4 months (95% CI: 15.1, 19.7). The 3-, 6-, 12-, 24- months overall survival (OS) rates were 93.7%, 90.0%, 76.8% and 64.3%, respectively, with a mean OS of 18.5 months (95% CI: 16.2, 20.7) (Figure 4).

Fifteen of the 39 CLM (38.5%) and 7 of the 24 NCLM (29.2%) had recurrence during follow-up. There was no significant difference in terms of recurrence between the two groups (p = 0.452). Moreover, 12 of the 46 liver metastasis with a tumor size of ≤3 cm (26%) and 10 of the 17 liver metastasis with a tumor size of >3 cm (58.8%) had recurrence after MWA. The recurrence rates were significantly higher in liver metastasis with a tumor size of >3 cm than in lesions with a tumor size of ≤3 cm (p < 0.001) (Table 3).

## 4. Discussion

Parenchyma-preserving methods are increasingly adopted in the primary or metastatic cancers of the liver [18]. Although there are studies in the literature reporting that partial hepatectomy offers better OS and DFS than thermal ablation methods, it should be considered that the bias caused by the performing of thermal ablation methods on patients who are not suitable for surgery may influence the results [19]. Also, surgical and thermal ablation methods have provided similar OS and DFS for recurrent liver metastases after partial hepatectomy [19]. Therefore, the prospective COLLISION trial results will provide a more accurate comparison of resection and ablation methods in similar lesions [20]. It has been reported that while MWA has similar clinical success to surgical resection in liver metastases, the complication rate is lower [21,22]. On the other hand, thermal ablation methods in patients not suitable for resection can provide a longer lifespan than chemotherapy alone [13,23]. In addition, MWA can make the lesion suitable for surgery by downstaging the initially unsuitable lesion [24,25].

Tilborg et al. revealed that MWA was effective in the treatment of unresectable CLM [26]. Moreover, Yuan et al. reported that thermal ablation was safe and effective for treating liver metastases of gynecological tumors [27]. Izzo et al. reported no significant difference between colorectal and noncolorectal metastases in recurrence and survival after MWA therapy [28]. Groeschl et al. reported that liver recurrence rates after MWA as 36.3% in CLM and 30.7% in NCLM and independent of tumor histology (except neuroendocrine tumor metastases), liver recurrences after MWA were closely related to tumor size and the number of tumors ablated [29]. In the current study, recurrence rates after MWA in the liver were 38.5% (15/39) in CLM and 29.2% (7/24) in NCLM, and it was higher in metastases with >3 cm. Li et al. reported that tumor diameter was an independent factor predicting LTP [30]. Therewithal, MWA is effective in liver metastases up to 5.5 cm [31]. It has been reported that the 1-, 2-year DFS and OS rates were 65.9%, 31.5%, and 81.8%, 60.8%, respectively, for MWA of hepatic metastases [30]. The current study revealed that the 1-, 2-year DFS and OS rates were 64.3%, 42.9%, and 77.5%, 61.6%, respectively, for MWA of hepatic metastases.

MWA emerged after RFA as a new therapy, and it is a safe and effective treatment method in primary and metastatic cancers of the liver [12]. It has been shown that MWA is superior to RFA in local tumor control in metastatic liver tumors [32,33]. However, no significant difference was found between RFA and MWA with regard to disease-free survival, local tumor progression, and sufficient ablation zone [34]. Remp et al. reported that LTP emerged at the edge of the ablation zone [35]. Therefore, successful ablation is only possible by properly positioning an appropriate antenna in the lesion, reaching an adequate temperature inside the lesion, and confirming that the ablation zone is sufficient. In this study, complete ablation was observed in all lesions in control CT one day after the ablation procedure. Previous research has addressed the importance of the safety margin of tumor ablation. In this study, the postprocedural ablation zone diameter ratio to preprocedural tumor diameter was evaluated; however, there was no significant difference between these rates.

In MWA, tumors located closer than 5 mm to critical organs, such as the intestinal system and great vessels, have a high risk for complications [16]. On the other hand, failure to create sufficient ablation zones to avoid complications during the ablation procedure of tumors in these localizations can reduce the technical success of the procedure and cause an increase in recurrence/residual tumor [36]. Different techniques have been used to overcome this issue, and the most common is hydrodissection. In the current study, hydrodissection was performed on 21 lesions, and none developed any complications. In the hydrodissection method, 0.9% saline or dextrose is percutaneously injected between the critical anatomical structure and the tumor. The ablation procedure is initiated after a sufficient safety gap is established [37]. Different commercial products, such as hyaluronic acid and poloxamer gel are also available for this purpose [38,39].

In malignant liver tumors, the major complication rate of MWA therapy has been reported to be 2.6%–2.9% [32,40]. Major complications are considered intestinal tract injury, major vessel injury, major biliary duct injury, bleeding requiring embolization, liver abscess, and tumor seeding. The minor complication rate of MWA therapy has been reported as 5.7%––7.3% [32]. The most common side effects related to the procedure are fever and general fatigue. In the current study, no major complication was observed, and the rate of minor complications (SIR classification grade A) was in accordance with the literature (6.4%).

This study has several limitations. First, the retrospective nature of this study might have influenced the results. Second, the sample size was also relatively small. Third, long-term follow-up results were lacking. There is a need for larger, prospective, and longer follow-up studies on this subject.

## 5. Conclusion

In conclusion, MWA therapy is as effective in the NCLM group as in the CLM group, regardless of histologic type. Metastasis size (>3 cm) was correlated with the recurrence rate in the CLM and NCLM groups.

## Figures and Tables

**Table 1:** Patient characteristics.

Variables	Total (47 patients with 63 lesions)	CLM (29 patients with 39 lesions)	NCLM (18 patients with 24 lesions)
Mean age (years)	59 ± 11.1	64.1 ± 8.9	50.7 ± 9.2
Sex (male/female)	25/22	22/7	3/15
Location of liver metastasis			
Near the larger vessels (n, %)	5 (7.9%)	3 (7.7%)	2 (8.3%)
Near gastrointestinal tract (n, %)	3 (4.8%)	3 (7.7%)	-
Near diaphragm (n, %)	6 (9.5%)	4 (10.2%)	2 (8.3%)
Near liver capsule (n, %)	5 (7.9%)	4 (10.2%)	1 (4.2%)
Near gallbladder (n, %)	2 (3.2%)	1 (2.6%)	1 (4.2%)
No special (n, %)	42 (66.7%)	24 (61.6%)	18 (75%)

CLM: colorectal liver metastases, NCLM: noncolorectal liver metastases, Near: closer than 5 mm

**Table 2:** Primary tumors of the patients.

	n	%
CLM		
Colon	20	42.5
Rectum	9	19.1
NCLM		
Breast	6	12.8
Gastric	4	8.5
Ovarian	3	6.4
Pancreas	3	6.4
Cervix	2	4.3

CLM: colorectal liver metastases, NCLM: noncolorectal liver metastases

**Table 3:** Comparison of recurrence rates by tumor type and size. 10 (58.8%)

	Tumor type		P* value	Tumor size		P* value
	CLM (39)	NCLM (24)		≤3 cm (46)	>3 cm (17)	
Recurrence (n, %)	15 (38.5%)	7 (29.2%)	0.452	12 (26%)	10 (58.8%)	<0.001

* Mann-Whitney U test,

CLM: colorectal liver metastases, NCLM: noncolorectal liver metastases
